# Endoscopic Anterolateral Ligament Reconstruction Using an Iliotibial Band Strip: A Cadaveric Feasibility Study

**DOI:** 10.7759/cureus.83992

**Published:** 2025-05-12

**Authors:** Théophile Stoclet, Pierre De Sulauze, Karam M Karam, Matthieu Lalevee, Olivier Courage

**Affiliations:** 1 Orthopedics and Traumatology, Centre Hospitalier Universitaire de Rouen, Rouen, FRA; 2 Orthopedics and Traumatology, Centre Hospitalier de Dieppe, Dieppe, FRA; 3 Orthopedics and Traumatology, Hopital privé de l'Estuaire - Ramsay Santé, Le Havre, FRA

**Keywords:** anterior cruciate ligament (acl) reconstruction, antero lateral ligament, kaplan fibers, knee surgery sports traumatology and arthroscopy, lateral extra articular tenodesis

## Abstract

Introduction

This study introduces an innovative, minimally invasive technique for reconstructing the anterolateral ligament of the knee. The approach involves harvesting a fascia lata band strip proximally to the lateral epicondyle (LE), thereby avoiding distal dissection. This method may replicate the function of Kaplan’s fibers by preserving a broad tibial insertion, potentially improving tibial rotational control. The aim of the study was to evaluate the feasibility of this technique under endoscopic guidance.

Materials and methods

The procedure was carried out on eight fresh-frozen cadaveric knees. The fascia lata strip was harvested endoscopically and fixed at the isometric point with the assistance of ultrasound guidance. Following the reconstruction, each knee was dissected to assess graft positioning.

Results

All eight procedures were successfully completed. Visualization of the fascia lata was consistently good throughout. The primary technical challenge was achieving adequate release of the deep plane, which was occasionally hindered by tissue adhesions. The harvested strips had a mean length of 44.7 mm (range: 38-51 mm) and a mean width of 10.9 mm (range: 9-13 mm). The femoral tunnel entry point was located an average of 8.2 mm (range: 7-10 mm) proximal and 5.2 mm (range: 4-6 mm) posterior to the LE.

Conclusions

This minimally invasive technique appears feasible under endoscopic guidance, enabling effective graft harvesting and precise positioning. By preserving a wide tibial insertion, the approach may enhance rotational control of the knee while minimizing surgical trauma.

## Introduction

The growing interest in the anterolateral ligament (ALL) stems from observations of persistent rotational instability following anterior cruciate ligament (ACL) reconstruction, as well as the high rate of graft retears seen in high-risk populations [1]. These findings underscore the importance of addressing the anterolateral structures to enhance knee stability and improve long-term surgical outcomes [2,3].

The ALL plays a crucial role in controlling internal tibial rotation [4] and serves a protective function for ACL grafts and meniscal repairs during the early stages of rehabilitation [5]. Consequently, combined ACL and ALL reconstruction is increasingly recommended, particularly in young or high-risk patients [6].

Although the anatomy of the ALL has been thoroughly described since the work of Claes et al. [7], replicating its isometric behavior during knee flexion remains a challenge in surgical practice [8]. Identifying the true isometric point, typically located superior and posterior to the lateral epicondyle (LE) [6], is technically demanding [9], especially given that the ALL does not maintain isometry throughout knee flexion [10,11]. In this context, intraoperative ultrasound may serve as a valuable tool to improve the precision of graft positioning and tunnel placement, thereby enhancing the reproducibility of ALL reconstructions [12].

Kaplan fibers, which are part of the deep layer of the anterolateral complex of the knee, contribute significantly to rotational stability and are frequently injured in ACL tears [13,14].

The surgical technique developed in this study is designed to preserve these fibers by using a proximally harvested fascia lata strip, thereby avoiding distal dissection to Gerdy’s tubercle. This minimally invasive modification of the original technique described by Christel and Djian [15] aims to replicate the function of the Kaplan fibers by maintaining a broad distal insertion of the fascia lata, which may improve rotational control [14]. The femoral isometric point was located using ultrasound guidance to enhance anatomical accuracy [12].

Contemporary trends in ligament reconstruction emphasize less invasive approaches that reduce postoperative pain, minimize soft tissue disruption, and offer better cosmetic results [16,17]. However, many current ALL reconstruction techniques still require distal dissection to Gerdy’s tubercle, resulting in larger incisions and a higher potential for postoperative discomfort.

The objective of this cadaveric study was to evaluate the feasibility of this endoscopic technique using two additional portals. Feasibility was defined by successful graft harvesting and accurate placement, while preserving key anatomical structures and minimizing surgical trauma.

## Materials and methods

This study was conducted in a university anatomical laboratory under an approved ethical protocol. Two knees were initially used to standardize the surgical technique and were excluded from the final analysis. The procedure was then performed on eight fresh-frozen cadaveric knees (demographic data unavailable), all dissected by a single experienced orthopedic surgeon. Specimens with a history of knee surgery or abnormal range of motion were excluded.

**Figure 1:**
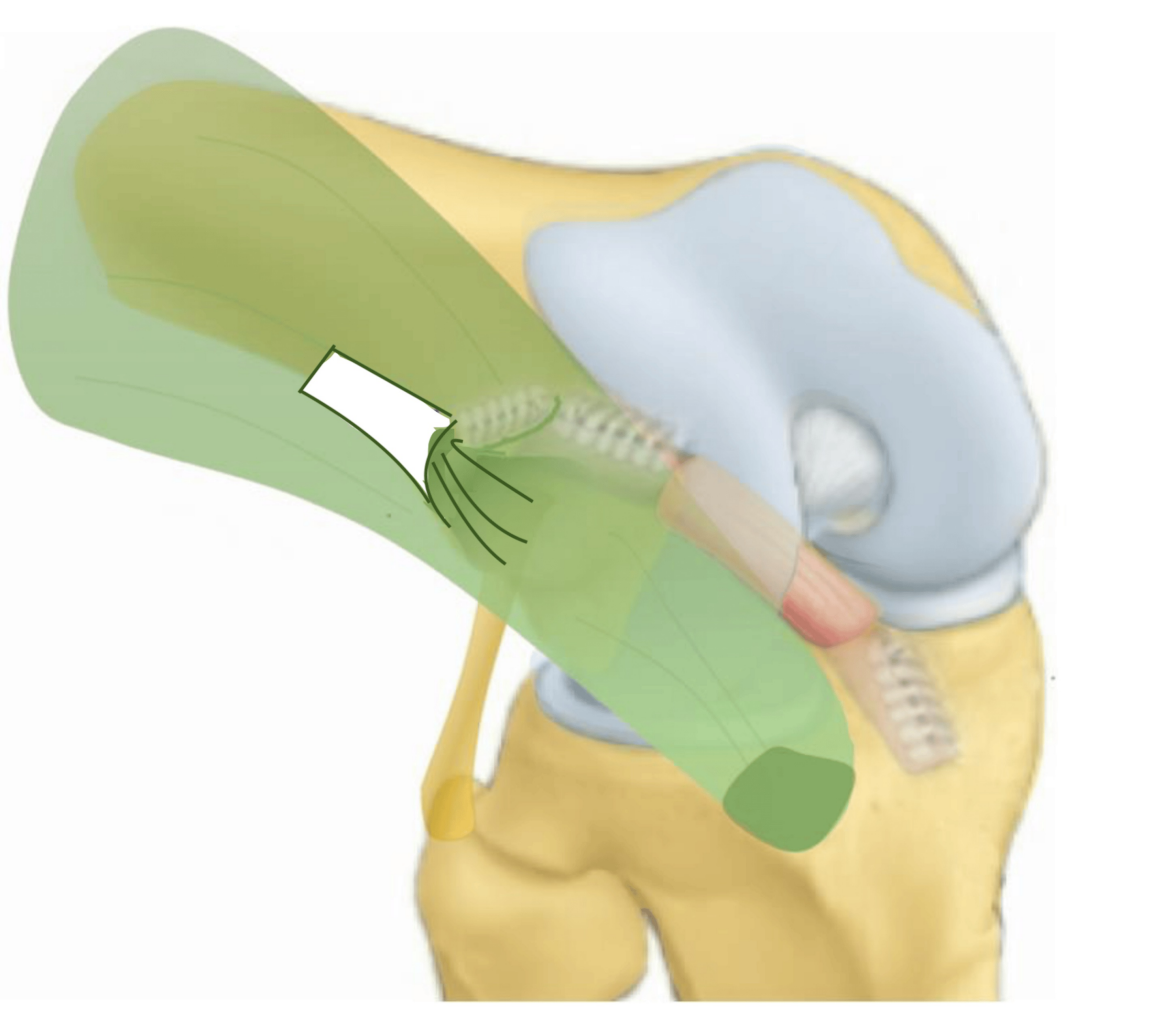
Graft illustration This novel technique is a modified version of the Christel-Djian method, as described by Courage et al. [18], utilizing an ITB strip dissected proximally to the LE without extending distally. This preserves a broader tibial insertion, potentially improving the distribution of rotational control and mimicking the function of Kaplan’s fibers. ITB, iliotibial band; LE, lateral epicondyle Permission for this figure was obtained from Springer Nature (license number 6007600286777).

The described technique was carried out (Figure 1), followed by open dissection to measure the femoral tunnel entry point in both anteroposterior and proximodistal planes relative to the LE in order to assess isometry. The graft was subsequently dissected to evaluate its length and width.

Surgical technique

Preoperative Setup

**Figure 2:**
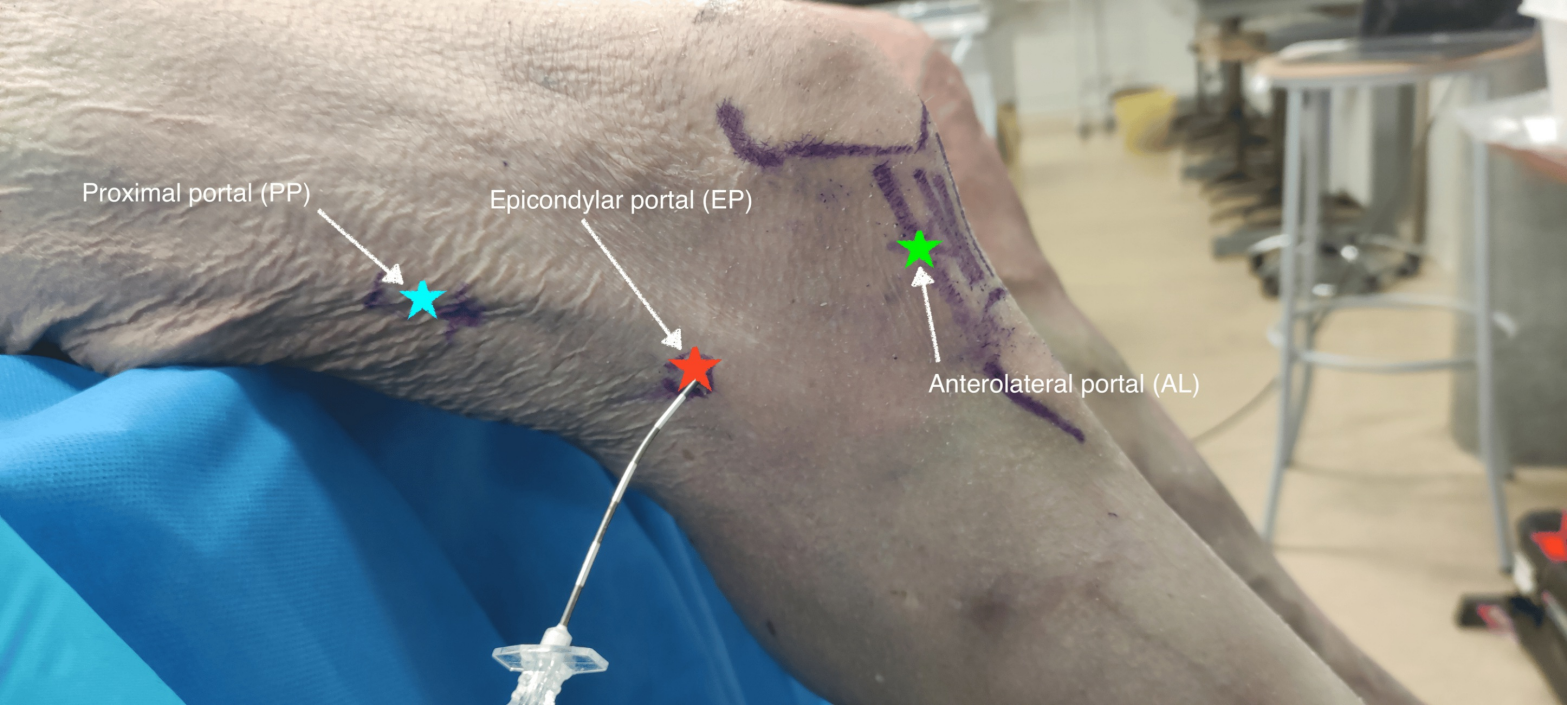
Marked endoscopic portals Three endoscopic portals were established: one at the level of the LE (EP), one located 5 cm proximally along the thigh axis (PP), and a standard AL. These portals were drawn and aligned on the skin. The EP was identified using ultrasound guidance. Knee flexion could be adjusted intraoperatively to optimize alignment of the three portals. AL, anterolateral portal; EP, epicondylar portal; LE, lateral epicondyle; PP, proximal portal

The cadaver was placed in a supine position with the knee flexed to 70° over the edge of the table. Anatomical landmarks, including the patella, patellar tendon, and planned surgical portals, were identified and marked (Figure 2).

The isometric point (KP) was defined according to Kittl et al. [19] and marked approximately 10 mm proximal and 8 mm posterior to the LE.

**Figure 3:**
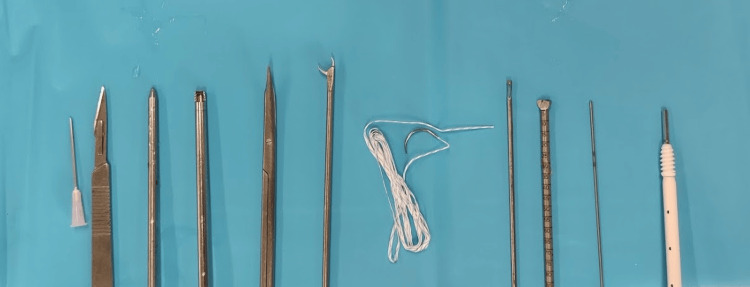
Arthroscopic set For this procedure, the following instruments were used: a needle for locating the EP, a no. 11 mm blade, a blunt trocar, a 4.5 mm shaver, slightly curved fine scissors, retrieval forceps, a no. 2 FiberWire with a tapered needle (Arthrex, Naples, FL, USA), a beath pin, a 6 mm reamer, a guide wire, and a 6.25 mm × 20 mm Bioswivelock (Arthrex). EP, epicondylar portal

Three portals were utilized for the procedure. The anterolateral portal (AL) served as the standard arthroscopic viewing portal. The epicondylar portal (EP) was positioned at the isometric point and marked using an ultrasound-guided needle fixed into the bone. The proximal portal (PP) was placed 5 cm proximal to the EP along the axis of the thigh. Standard arthroscopic instruments were prepared for the procedure (Figure 3).

Workspace Optimization

**Figure 4:**
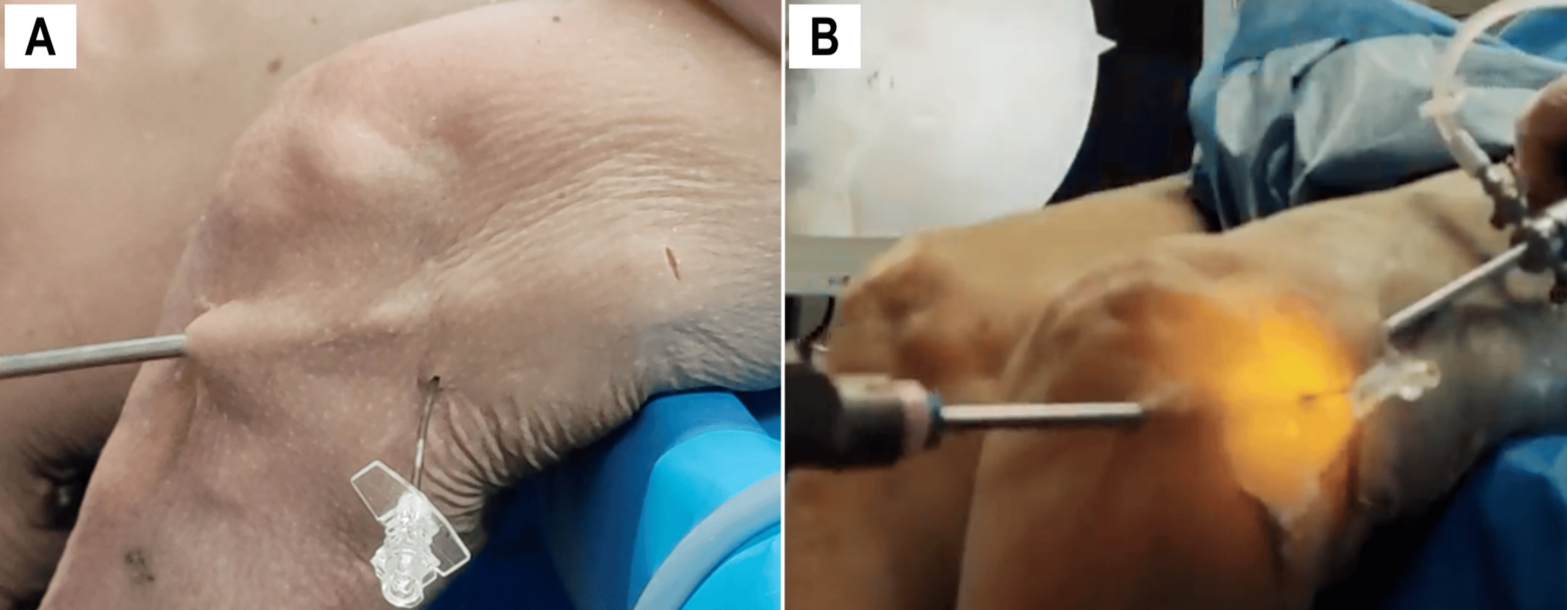
Working space creation External view of a left knee. A blunt trocar is used to create a subcutaneous space from the AL to the PP (A). A 30° arthroscope (inserted through the PP) and a 4.5 mm shaver (inserted through the AL) are then introduced (B). AL, anterolateral portal; PP, proximal portal

**Figure 5:**
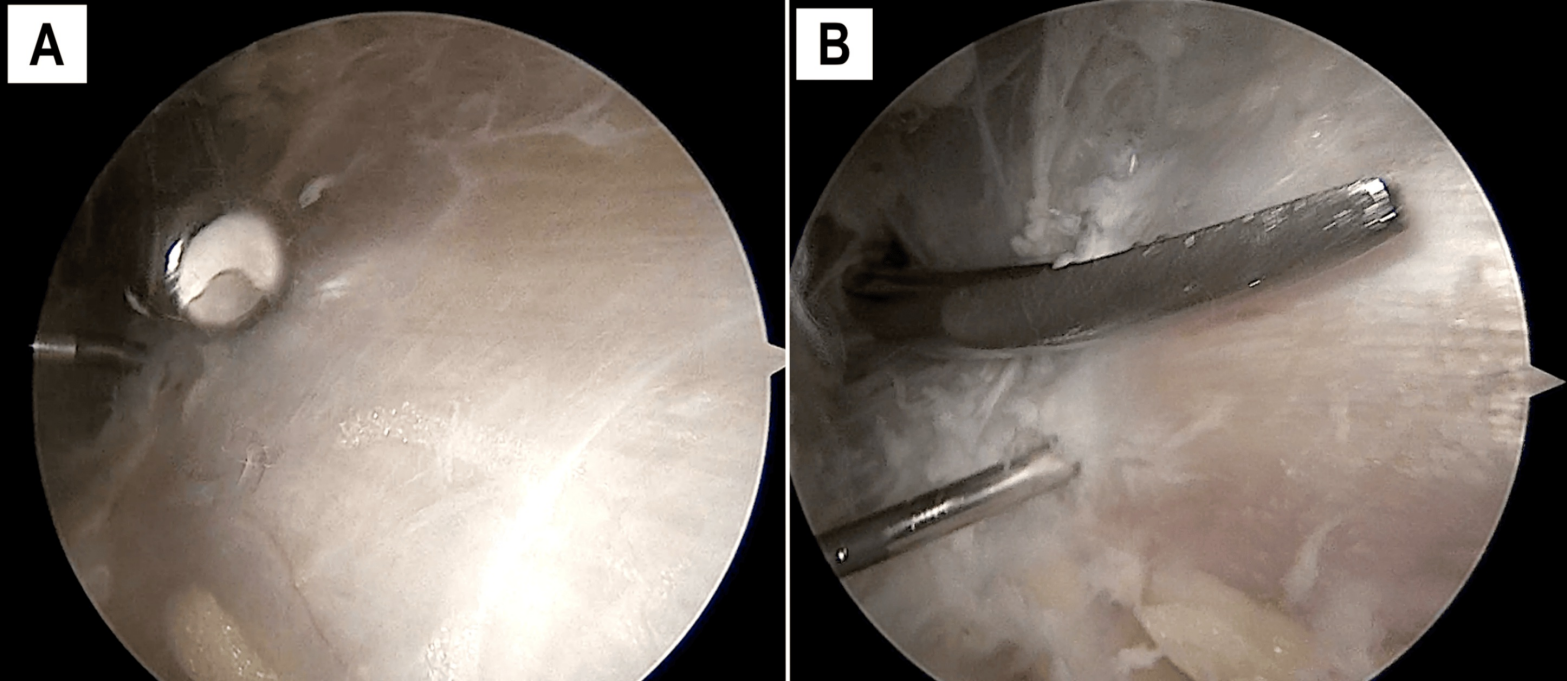
Graft section Arthroscopic views of a left knee, from the PP. A 4.5 mm shaver introduced through the AL is used to release the fascia lata from subcutaneous adhesions (A). The shaver is then replaced by curved fine scissors to perform the first longitudinal incision (B). The needle serves as a reliable landmark in both views (A, B). AL, anterolateral portal; PP, proximal portal

A blunt trocar was introduced through the AL to create a subcutaneous working space extending between the AL and PP (Figure 4A). A 4.5 mm shaver and a 30° arthroscope were inserted through the PP (Figure 4B). The fascia lata was then released from any subcutaneous adhesions (Figure 5A).

Iliotibial Band (ITB) Strip Preparation

**Figure 6:**
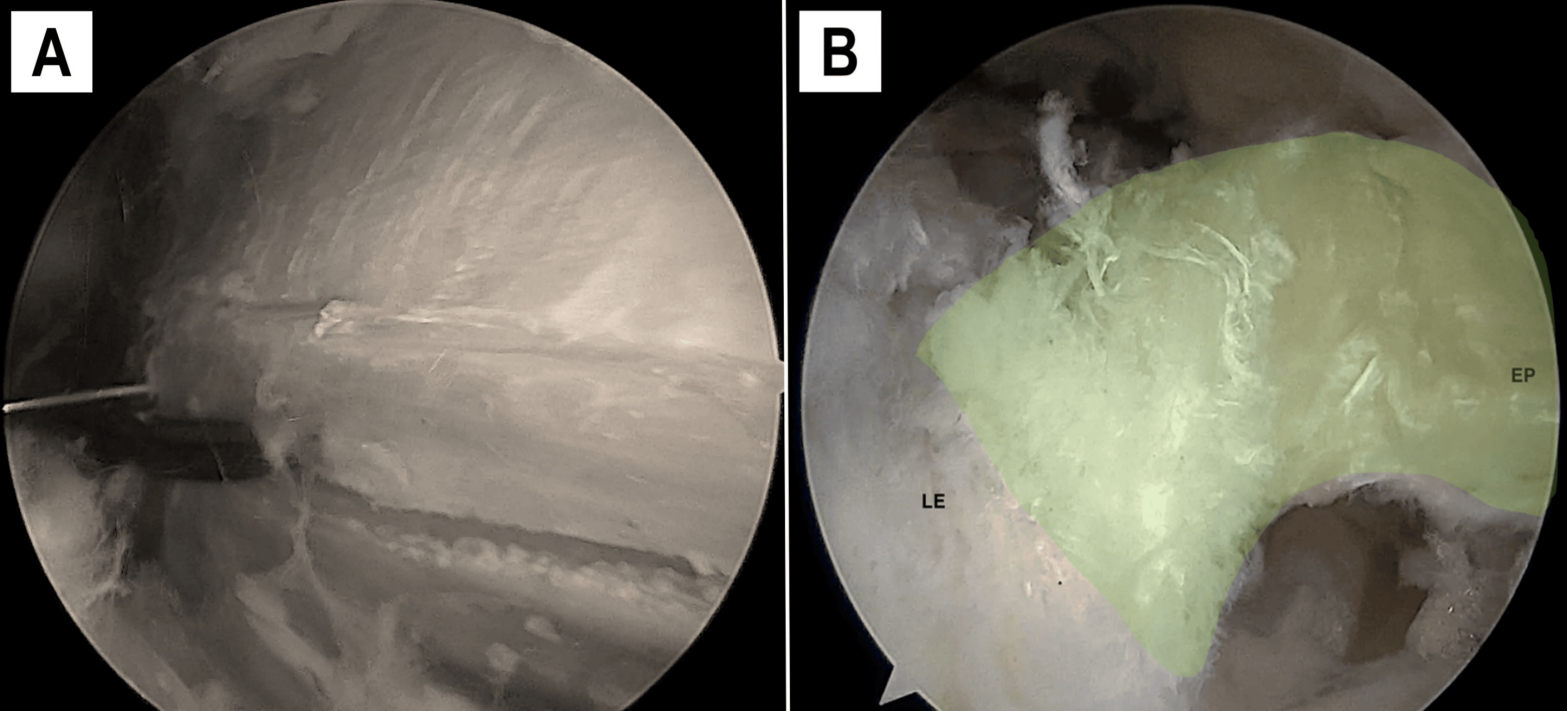
Graft release Arthroscopic view of a left knee from the PP (A) showing the second parallel incision on the opposite side of the needle. The portals are then switched, and the strip is detached proximally. The anterolateral view (B) shows the released fascia lata band strip (highlighted in green) free of any adhesions. PP, proximal portal

With the knee flexed at 90° to optimize alignment with the fibers of the fascia lata, fine curved scissors were inserted through the AL. Two parallel incisions were made on the fascia lata towards the PP, guided by the needle’s entry point (Figure 5B, Figure 6A). Endoscopic guidance ensured precise control over the length and positioning of the incisions. The strip was approximately 10 mm in width (5 mm on either side of the needle) and measured between 40 and 50 mm in length. Prior to releasing the proximal end, the strip was carefully detached from the subfascial plane.

The arthroscope and scissors were then switched between portals, and the graft was detached by cutting the proximal attachment approximately 4-5 cm from the needle (Figure 6B). Once the needle was removed, the EP was established.

**Figure 7:**
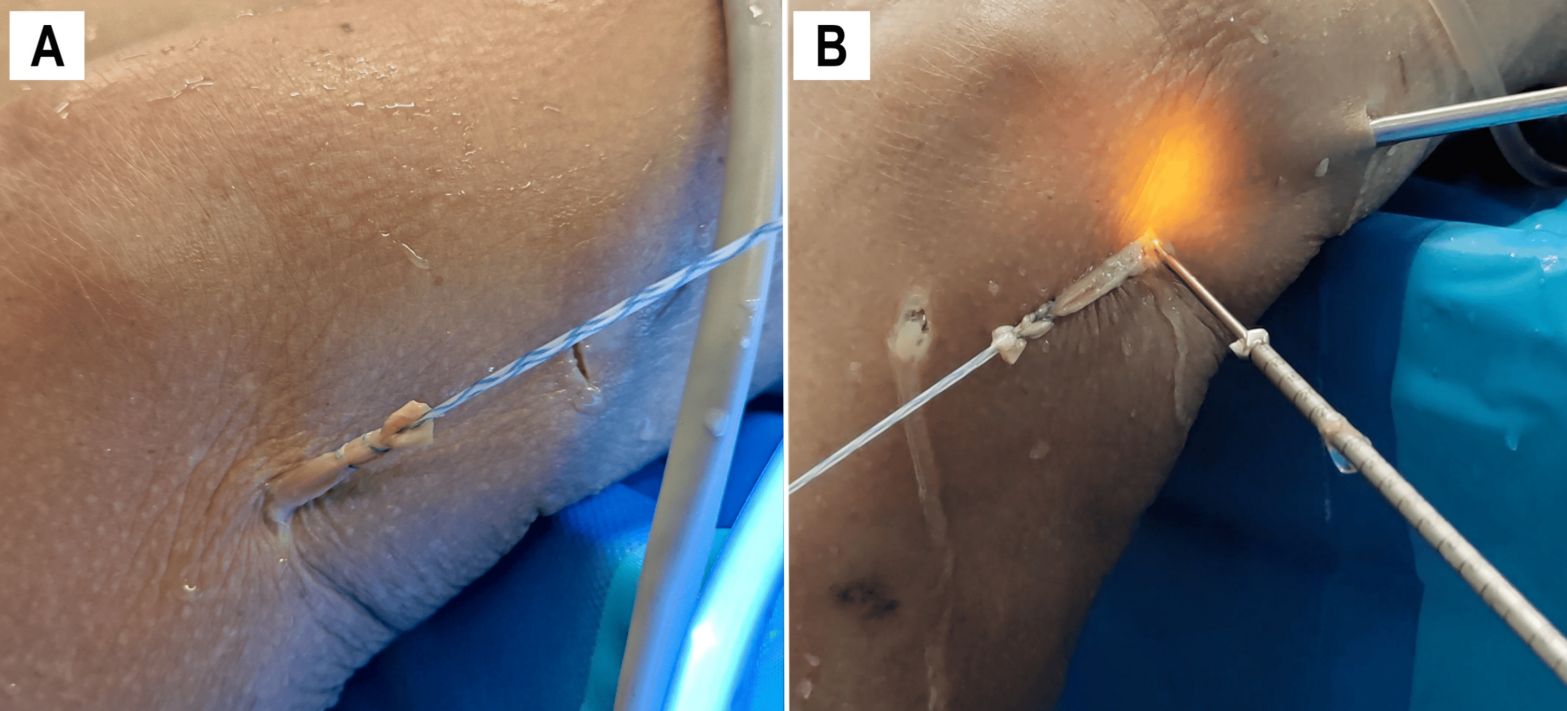
Graft and femoral tunnel preparation External views of a left knee. After removing the needle, the fascia lata graft is exteriorized through the EP and reinforced with a FiberLoop (A). A beath pin is inserted into the femur at the isometric point to guide the drilling of a 50 mm tunnel (B). EP, epicondylar portal

The graft was then externalized through the EP using a retrieval forceps. It was measured, reinforced over 15 mm using a FiberLoop (Arthrex, Naples, FL, USA), and calibrated (Figure 7A). The external segment of the graft measured 3-4 cm, with approximately 1 cm designated for intraosseous insertion.

Femoral Preparation

With distal tension applied to the graft, a beath pin was inserted proximal to the graft root under endoscopic visualization. It was externalized anteromedially, just above the patella, in accordance with the recommendations of Smeets et al. to avoid interference with the ACL tunnel [20]. A 6 mm cannulated compaction reamer was then used to drill a 50 mm femoral tunnel (Figure 7B).

Graft Fixation and Tensioning

**Figure 8:**
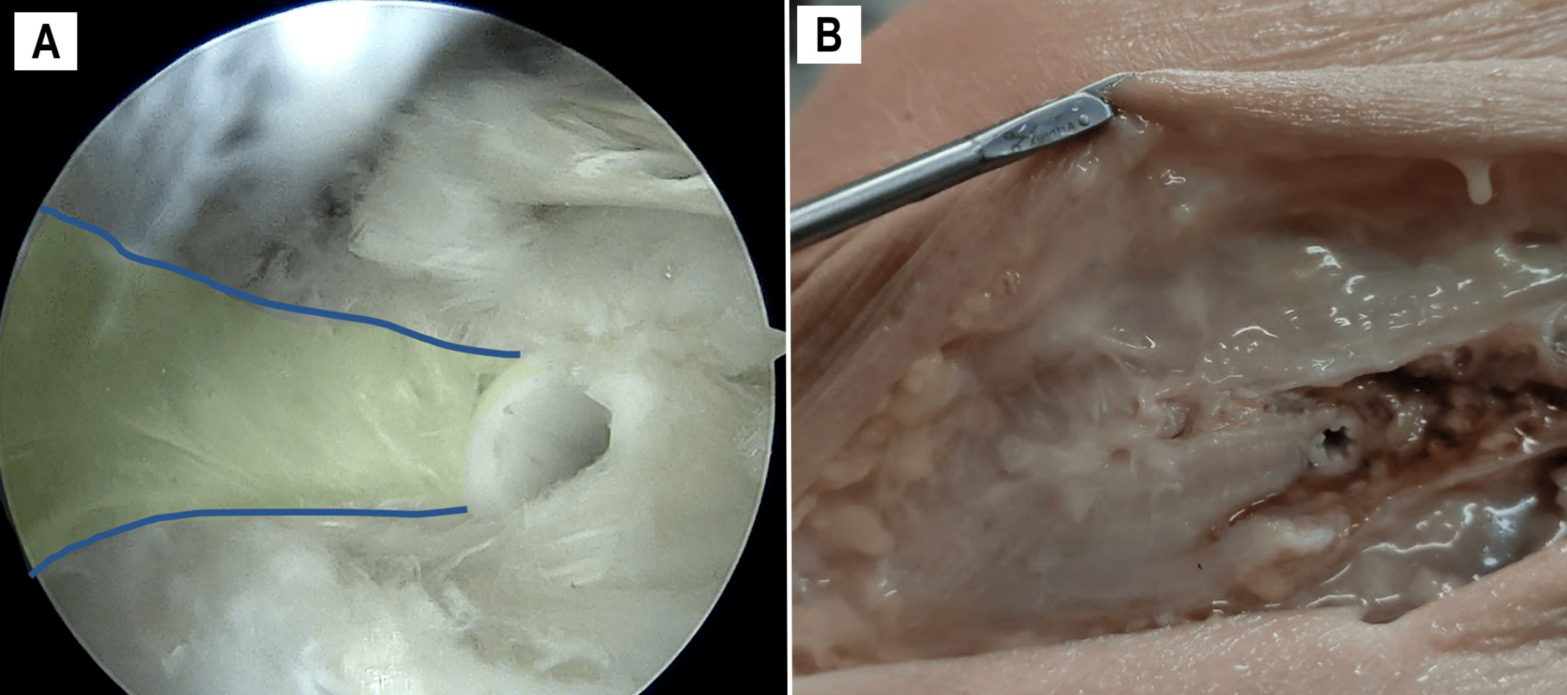
Graft final aspect (endoscopic and open view) Final views of the reconstruction after insertion into the femoral tunnel and fixation with a 6.25 mm Bioswivelock (Arthrex, Naples, FL, USA). (A) Arthroscopic view from the PP (graft highlighted in green). (B) Open view of the reconstruction after dissection. PP, proximal portal

The FiberLoop suture was passed through the beath pin. With the knee flexed at 30° in a neutral rotational position, a guide wire was introduced, and the graft was tensioned within the femoral tunnel. A 6.25 mm × 20 mm interference screw was inserted to secure the graft (Figure 8A). Fixation and tensioning were verified by applying internal rotational stress at 30° of flexion. Subsequently, an open dissection was performed with the knee flexed at 70°, along with dynamic testing to evaluate the reconstruction (Figure 8B). The full surgical procedure is detailed in Video 1, and the key steps along with technical tips are summarized in Table 1.

**Video 1:** Surgical technique This video demonstrates a novel lateral extra-articular tenodesis technique using a fascia lata strip harvested without distal dissection, performed entirely under endoscopic visualization.

**Table 1:** Surgical steps and tips AL, anterolateral portal; EP, epicondylar portal; LE, lateral epicondyle; PP, proximal portal

Key surgical steps	Instruments (portals)	Tips
1. Identify the LE and insert a needle slightly posterior and proximal to mark the EP.	Needle (EP)	Ultrasound can assist in optimal localization.
2. Identify the PP, located 50 mm proximal to the EP.	-	-
3. Create a subcutaneous tunnel between the AL and PP.	Blunt trocar (AL)	-
4. Release the fascia lata from subcutaneous adhesions.	Shaver (AL), scope (PP)	The needle serves as a helpful anatomical landmark.
5. Make two parallel incisions along the fascia lata fibers, extending from distal to proximal.	Scissors (AL), scope (PP)	Varying the degree of knee flexion can help align with the fiber direction.
6. Cut the proximal attachment approximately 50 mm from the needle.	Scissors (PP), scope (AL)	Ensure the strip is fully detached from the subfascial plane beforehand.
7. Externalize the fascia lata strip through the EP and reinforce it with a FiberLoop.	Forceps (EP), scope (AL)	-
8. Insert a beath pin at the isometric point.	Beath pin (EP), scope (AL)	-
9. Ream a 50 mm femoral tunnel using a 6 mm reamer.	Reamer (EP), scope (AL)	-
10. Pull the FiberLoop suture through the tunnel using the beath pin.	Scope (AL)	-
11. Introduce a guide wire into the femoral tunnel.	Scope (AL)	-
12. Tension the graft and confirm appropriate positioning and tensioning.	Scope (AL)	Maintain neutral rotation with the knee flexed at 30°.
13. Fix the graft using a 6.25 mm × 20 mm interference screw over the guide wire.	Scope (AL)	The knee should remain at 30° flexion in neutral rotation
14. Remove the guide wire and test graft stability by applying internal rotational stress.	Scope (AL)	-

## Results

**Figure 9:**
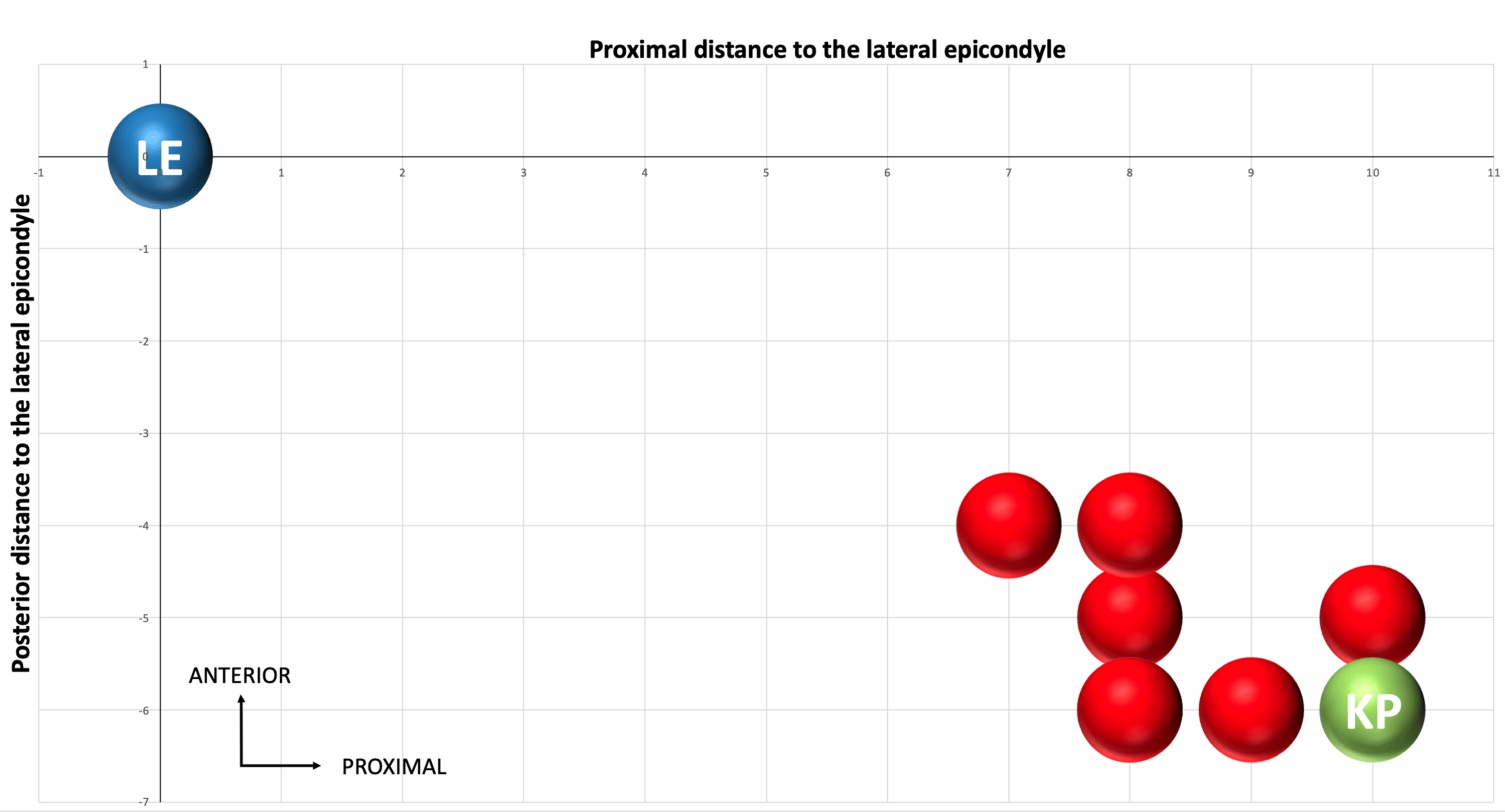
Graphical representation of the tunnels’ relative position Illustration showing the location of the created femoral tunnels (red) in relation to the target isometric point (KP, green) and the LE (blue). All measurements are in millimeters. The tunnels were positioned slightly distal and anterior to the target, but still within the acceptable isometric region. LE, lateral epicondyle

Two cadaveric knees were initially used to refine the surgical technique and were excluded from the primary analysis, which was subsequently performed on eight additional cadaveric knees. In all cases, visualization and release of the fascia lata were consistently achieved without difficulty. Creating the two parallel sections was straightforward; however, fluid leakage occasionally occurred due to the insertion of the scissors. Proximal graft sectioning was facilitated by the initial release, aided by the natural tension of the tissue. No macroscopic injuries to the iliotibial tract, adjacent musculature, or neurovascular structures were observed during or after dissection. Graft measurements were recorded for each specimen (see Appendix A), with mean values presented in Table 2. A schematic illustration of the tunnel placement for each knee is shown in Figure 9.

**Table 2:** Description of global femoral tunnel length, width, and position Post. LE: posterior distance of the tunnel from the LE; Prox. LE: proximal distance of the tunnel from the LE LE, lateral epicondyle

Variable	Mean	Median	Min-max	95% CI
Length (mm)	44.75	45.5	38.00-51.00	40.790-48.710
Width (mm)	10.88	11	9.00-13.00	9.735-12.015
Post. LE (mm)	5.25	5.5	4.00-6.00	4.557-5.943
Prox. LE (mm)	8.25	8	7.00-10.00	7.557-8.943

## Discussion

The cadaveric findings confirm the technical feasibility of an endoscopic-assisted ALL reconstruction using a proximally harvested ITB strip. This technique aligns with the current shift toward minimally invasive surgical approaches [16,17].

Various ALL reconstruction techniques have been previously described, often involving tendon harvesting, such as gracilis autografts, to enhance rotational control [3,4]. In contrast, our approach offers a minimally invasive alternative by utilizing a fascia lata strip, a well-established graft in ligament reconstruction. Unlike the technique reported by Christel and Djian [15], this method avoids distal dissection of Gerdy’s tubercle, preserving a broad tibial insertion and thereby better replicating the function of Kaplan’s fibers. These fibers anchor the ITB to the posterolateral distal femur and are essential for rotational stability [14]. Since Kaplan’s fibers are frequently damaged in ACL injuries [13], restoring their function may improve postoperative knee stability [14].

Adapting this procedure to an endoscopic technique offers several advantages. The use of the standard AL with two additional small incisions minimizes surgical morbidity. This approach eliminates the need for a tibial tunnel, preserving bone stock and avoiding potential tunnel conflicts. Furthermore, identifying the isometric point with an ultrasound-guided needle offers a reliable and reproducible method for accurate tunnel placement [12].

Technical considerations and challenges

Some technical challenges were encountered during the procedure. The use of fine scissors for graft harvesting led to fluid leakage and pressure loss in the endoscopic field. This could be improved with dedicated arthroscopic scissors or specialized tools to allow for better control. Aligning the incisions with the ITB fiber orientation also required adjusting knee flexion intraoperatively to optimize tension and visualization. A standardized positioning protocol may help address this issue.

Graft quality and positioning

The harvested graft dimensions were generally consistent, although in two cases the strip length was below 40 mm. While this did not prevent reconstruction, shorter grafts may reduce pull-out strength, an aspect not evaluated in this study. Future biomechanical testing should assess whether variations in graft size affect fixation strength and rotational stability.

Tunnel positioning, guided by ultrasound, resulted in an average tunnel entrance 8.2 mm proximal and 5.2 mm posterior to the lateral epicondyle. Although the target coordinates were 10 mm and 6 mm, respectively, these slight deviations still fell within the accepted isometric zone [6,9]. The functional implications of minor discrepancies in tunnel placement remain unclear, as a universally defined “perfect” isometric point has yet to be established [10,11].

Limitations

This study was conducted on cadaveric specimens, which introduces several inherent limitations. Potential complications such as bleeding, healing response, and clinical stability could not be evaluated and require investigation in live subjects. Additionally, the clinical implications of subcutaneous detachment must be assessed in future studies. Finally, the biomechanical strength of the harvested grafts was not precisely measured, limiting the ability to extrapolate these findings directly to clinical practice.

## Conclusions

This cadaveric study demonstrates the feasibility of an endoscopic-assisted ALL reconstruction technique using a proximally harvested ITB strip. Ultrasound guidance proved effective for accurately locating the femoral isometric point, resulting in consistent tunnel positioning. The technique preserved the natural tibial insertion of the ITB and avoided distal dissection, thus maintaining the functional contribution of Kaplan’s fibers and enhancing tibial rotational control. This minimally invasive, tissue-preserving approach also reduces donor-site morbidity and scarring. These findings suggest the potential of this technique as a valuable option for ALL reconstruction. However, further biomechanical and clinical studies are needed to validate its long-term efficacy and functional outcomes.

## Appendices

Appendix A

**Table 3:** Measurements of femoral postion after dissection for each knee Post. LE: posterior distance of the tunnel from LE; Prox. LE: proximal distance of the tunnel from LE LE, lateral epicondyle

Knee N°	Length (mm)	Width (mm)	Post. LE (mm)	Prox. LE (mm)
1	38	9	5	8
2	44	12	4	7
3	38	12	4	8
4	47	10	6	8
5	49	11	6	8
6	51	9	6	8
7	42	11	5	10
8	49	13	6	9

## References

[1] Smith PA, Bley JA (2016). Minimally invasive anterolateral ligament reconstruction of the knee. Arthrosc Tech.

[2] Sonnery-Cottet B, Saithna A, Cavalier M (2017). Anterolateral ligament reconstruction is associated with significantly reduced ACL graft rupture rates at a minimum follow-up of 2 years: a prospective comparative study of 502 patients from the SANTI Study Group. Am J Sports Med.

[3] Helito CP, Sobrado MF, Giglio PN, Bonadio MB, Pécora JR, Camanho GL, Demange MK (2019). Combined reconstruction of the anterolateral ligament in patients with anterior cruciate ligament injury and ligamentous hyperlaxity leads to better clinical stability and a lower failure rate than isolated anterior cruciate ligament reconstruction. Arthroscopy.

[4] Sonnery-Cottet B, Lutz C, Daggett M, Dalmay F, Freychet B, Niglis L, Imbert P (2016). The involvement of the anterolateral ligament in rotational control of the knee. Am J Sports Med.

[5] Sonnery-Cottet B, Saithna A, Blakeney WG (2018). Anterolateral ligament reconstruction protects the repaired medial meniscus: a comparative study of 383 anterior cruciate ligament reconstructions from the SANTI Study Group with a minimum follow-up of 2 years. Am J Sports Med.

[6] Sonnery-Cottet B, Daggett M, Fayard JM (2017). Anterolateral Ligament Expert Group consensus paper on the management of internal rotation and instability of the anterior cruciate ligament - deficient knee. J Orthop Traumatol.

[7] Claes S, Vereecke E, Maes M, Victor J, Verdonk P, Bellemans J (2013). Anatomy of the anterolateral ligament of the knee. J Anat.

[8] Imbert P, Lutz C, Daggett M, Niglis L, Freychet B, Dalmay F, Sonnery-Cottet B (2016). Isometric characteristics of the anterolateral ligament of the knee: a cadaveric navigation study. Arthroscopy.

[9] Yao G, Liu Y, Zhou Z (2024). A cadaveric study of the optimal isometric region on the anterolateral surface of the knee in anterolateral ligament reconstruction. Orthop Surg.

[10] Helito CP, Helito PV, Bonadio MB (2014). Evaluation of the length and isometric pattern of the anterolateral ligament with serial computer tomography. Orthop J Sports Med.

[11] Zens M, Niemeyer P, Ruhhammer J (2015). Length changes of the anterolateral ligament during passive knee motion: a human cadaveric study. Am J Sports Med.

[12] Cavaignac E, Castoldi M, Marot V, Courtot L, Gracia G, Reina N (2019). Minimally invasive ultrasound-guided anterolateral ligament reconstruction with autologous 2-strand gracilis graft. Arthrosc Tech.

[13] Marom N, Greditzer HG 4th, Roux M, Ling D, Boyle C, Pearle AD, Marx RG (2020). The incidence of Kaplan fiber injury associated with acute anterior cruciate ligament tear based on magnetic resonance imaging. Am J Sports Med.

[14] Ayati Firoozabadi M, Mortazavi SM, Shakiba M (2025). Investigating injuries to the anterolateral corner of the knee: imaging insights into kaplan fibers and the anterolateral ligament in anterior cruciate ligament tears. Orthop J Sports Med.

[15] Christel P, Djian P (2002). Anterio-lateral extra-articular tenodesis of the knee using a short strip of fascia lata [Article in French]. Rev Chir Orthop Reparatrice Appar Mot.

[16] Sonnery-Cottet B, Barbosa NC, Tuteja S, Daggett M, Kajetanek C, Thaunat M (2016). Minimally invasive anterolateral ligament reconstruction in the setting of anterior cruciate ligament injury. Arthrosc Tech.

[17] Imbert P (2007). Minimally invasive extra-articular anterolateral reinforcement: a new technique. Arthroscopy.

[18] Courage O, Bertiaux S, Papin PE, Kamel A (2021). Lateral tenodesis: extra-articular reconstruction with the fascia lata using a modified Christel-Djian technique. Knee Arthroscopy.

[19] Kittl C, Halewood C, Stephen JM, Gupte CM, Weiler A, Williams A, Amis AA (2015). Length change patterns in the lateral extra-articular structures of the knee and related reconstructions. Am J Sports Med.

[20] Smeets K, Van Haver A, Van den Bempt S (2019). Risk analysis of tunnel collision in combined anterior cruciate ligament and anterolateral ligament reconstructions. Knee.

